# Questioning origins: the role of ethical and metaethical claims in the debate about the evolution of morality

**DOI:** 10.1007/s13194-025-00635-7

**Published:** 2025-02-13

**Authors:** Rebekka Hufendiek

**Affiliations:** Humboldt-Zentrum Für Philosophie Und Geisteswissenschaften, Universität Ulm, Albert-Einstein-Allee 5, 89081 Ulm, Germany

**Keywords:** Evolution of morality, Moral psychology, Pluralism, Values in science, Mixed claims

## Abstract

Research about the evolution of morality suffers from the lack of a clear, agreed-upon concept of morality. In response to this, recent accounts have become increasingly pluralist and pragmatic. In this paper, I argue that 1) both the concept of morality and the broader understanding of what makes us moral include ethical and metaethical assumptions; 2) there is no uncontroversial descriptive notion available, and therefore settling on a particular concept inevitably entails such assumptions; and 3) what is lacking is a reflection on the role that ethical and metaethical assumptions play, suggesting that the debate would benefit from making them explicit. Claims about “the true origin of morality” can fruitfully be analyzed as “mixed claims”: claims that combine a causal-historical hypothesis (e.g., about the evolution of a certain ability, such as empathy or joint intentionality) with ethical or metaethical assumptions about which abilities or norms make us moral. Making such assumptions explicit advances the epistemic aims of transparency and comparability, and thereby helps to avoid rash conclusions regarding, for instance, the nature of moral progress. Finally, it helps to unpack the normative knowledge shared by behavioral scientists and comparative psychologists and to give this knowledge its proper place in research.

## Introduction

Recent years have seen the publication of numerous books and articles on the evolution of human morality by behavioral scientists, comparative psychologists, and philosophers (Churchland, 2011; Curry et al., 2019; Kitcher, 2011; Kumar & Campbell, 2022; Tomasello, 2016). Even a cursory glance at this literature reveals a range of disparate narratives about the emergence of morality. Did the evolution of caring behavior turn us into moral creatures? Or was it emotions such as guilt and shame? Or did the evolution of social cognitive abilities such as joint intentionality do it? Or could it have been some combination of these factors?

This lack of consensus has resulted in growing doubt that the concept of morality picks out a natural kind: a cluster of psychological properties that are essential for morality (Machery & Stich, 2022; Stich, 2019; Westra & Andrews, 2022). In response to this doubt, some researchers have moved away from morality as an explanandum, suggesting that it might prove more fruitful to look for normative regularities, or basic normative abilities, found in infants and, to some degree, in other species (Andrews, 2020; O’Neill, 2017; O’Neill & Machery, 2019; Westra & Andrews, 2022).

I argue in this paper that 1) both the concept of morality and the broader understanding of what makes us moral include ethical and metaethical assumptions, i.e. different concepts of morality are tied to specific ethical and metaethical theories; 2) there is no uncontroversial descriptive notion available, and therefore settling on a particular concept—even for pluralists or pragmatists— inevitably entails such assumptions; and 3) what is lacking is a reflection on the role that ethical and metaethical assumptions play, suggesting that the debate would benefit from making them explicit. As a result, claims about when and how the evolution of morality occurred are mixed claims. Settling on a particular concept of morality, or adopting a broader understanding of what makes us moral, commits the author to ethical or metaethical assumptions—even if that concept is chosen for pragmatic reasons such as methodological feasibility. Accordingly, when authors in the field make claims such as “the first sparks of morality were feelings of sympathy and loyalty that arose between kin and friends” (Kumar & Campbell, 2022, p. 34) or “The cooperative rationality we are positing here is the ultimate source of the human sense of ‘ought’” with this in place “were born genuinely, if only locally, moral beings.” (Tomasello, 2016, p. 82–83), it is productive to analyze these as mixed claims (Alexandrova, 2018). Mixed claims involve concepts that rely on a normative standard. Many such concepts, including “wellbeing” and “health,” play central roles in scientific research. Empirical generalizations about these concepts require normative assumptions. I argue that “morality” is such a concept that unavoidably contains ethical or metaethical background assumptions.

Likewise, a claim such as that by Tomasello about “the ultimate source of ought” combines a causal-historical hypothesis (e.g., about the evolution of a certain ability, such as empathy or joint intentionality) with a normative assumption about which abilities make us moral. I argue that the debate about the evolution of morality would benefit from making the normative dimension in mixed claims explicit and negotiable.

In the second section of this paper, I introduce the concept of mixed claims in science and apply it to morality. In the third section, I offer a representative overview of the current literature on the evolution of morality to show that there is disunity in how the concept of morality is understood, and that it is imbued with ethical and metaethical assumptions. The fourth section demonstrates that neither philosophy nor the empirical sciences can settle on a concept of morality that is joint-carving and free from ethical or metaethical assumptions. In the fifth section, I show how disunity tends to result in implicit mixed claims, which lead to biased or even fallacious conclusions. In the final section, I discuss how making mixed claims explicit can be productive. I argue that authors should invest effort in discussing how their accounts relate to others to ensure comparability. Rather than limiting these comparisons to empirical differences; instead, they should negotiate possible disagreements about ethical and metaethical assumptions as such.

## Mixed claims in science

Several concepts that play central roles in scientific research, such as “wellbeing” and “health,” have definitions that rely on normative standards. Anna Alexandrova has introduced the notion of a “mixed claim” for claims that relate an empirical variable to a variable that is defined in partly normative terms. A hypothesis is mixed if and only if (1) it is an empirical hypothesis about a putative causal or statistical relation and (2) at least one of the variables in it is defined in a way that presupposes a value judgment about the nature of this variable, where the relevant value is not an epistemic or cognitive one, such as coherence or empirical adequacy (since these do not threaten the value free ideal) but rather a moral, prudential, political, or aesthetic value (Alexandrova, 2018, 2021, p. 82).

Empirical claims about wellbeing, according to Alexandrova, have such a structure. Consider the claim that “Living in East Saint Louis harms wellbeing.” This relates a purely descriptive variable—geographic location—with a variable—wellbeing—defined in partly normative terms.^1^ What is meant by “wellbeing” can significantly differ from one study to another. In psychology alone, there are three ways of using the concept.^2^ The first treats wellbeing as reported happiness, the second as reported overall life satisfaction, and the third as flourishing or functioning well. Each is grounded in different background assumptions about what wellbeing is. These assumptions are partly theoretical—for instance, insofar as they understand wellbeing to be constituted by subjective (happiness or overall life satisfaction) versus objective (flourishing) measures. But these theoretical differences come combined with normative differences: some authors suggest that a life satisfaction notion of wellbeing best respects the judgment of the individual (Diener et al., 2009), while others argue that only measures based on objective quality of life can bring inequalities and injustices within societies into view (Nussbaum & Sen, 1993).

In empirical research, a concept of wellbeing is typically chosen not with regard to theoretical or normative factors, but rather with regard to prudential factors such as the feasibility of the method. For a statement to be a mixed claim, however, its normative assumptions need to be made neither explicitly nor intentionally. When researchers ask whether “commuting harms wellbeing,” and chose a relevant concept of wellbeing for solely methodological reasons, this decision still carries a normative dimension that will influence the result and that can be debated on normative grounds. A staunch Aristotelian about wellbeing, who believes that only the virtuous can flourish, for example, will miss a morality constraint in the measure of wellbeing that focuses on happiness alone.

I propose that claims about the evolution of morality are a specific case of mixed claims. Because we lack an uncontroversial descriptive concept of morality, claims about the evolution of morality cannot simply be descriptive or causal-historical claims about what happened. The claim I want to establish is stronger than the claim that the choice of a concept of morality (or any other concept) is partly normative in the sense that there will be alternative ways to define the concept, each having advantages and disadvantages, and that the theorist has to make an all-things-considered judgment as to which concept is best overall. Such a choice might rest entirely on epistemic values, such as empirical adequacy or consistency. As will become clear in the following sections, I think that the choice of a concept of morality inevitably entails ethical or metaethical claims—for instance, claims about which values are morally relevant or which abilities make us moral agents.

Why does this matter? If accounts of the evolution of morality differ substantially in what they take morality to be, and if the differences inevitably commit the authors to ethical and metaethical assumptions that are not fully explicit, then the epistemic aims of transparency and comparability will suffer. I return to this problem in the final section.

## Conceptual disunity in the debate about the evolution of morality

In this section, I make the conceptual disunity in the recent literature on the evolution of morality explicit, so that the next section can examine the normative implications of the different concepts more closely. There is considerable disunity among authors’ concepts of morality. One reason is that it is an interdisciplinary research field: primatologists and behavioral scientists tend to focus on prosocial behaviors, psychologists on moral emotions and cognition, and philosophers on practical reasons. However, we shall see that this captures only part of a much deeper disunity. The differences between concepts of morality can be characterized along two dimensions. The first is the spectrum of abilities considered constitutive for someone to be a moral agent, ranging from abilities which lead to prosocial behaviors to those involving practical reasoning. Authors who construct a narrative about the evolution of morality must decide which abilities are relevant—specifically, which abilities needed to evolve for morality to evolve. Some authors make explicit choices by arguing that the ability to show altruistic behavior is not yet a central moral ability, while others make implicit choices, such as omitting emotions from their narrative without explicitly arguing against sentimentalism.^3^ The second dimension concerns the spectrum of possible criteria for distinguishing moral norms from conventional norms, with reference to values or formal features such as objectivity, universality, or categoricity. I will argue that constructing a narrative on the evolution of morality requires taking a stance on questions such as: Which values are moral values and which are social or conventional? Are moral values universalizable or categorical? What distinguishes moral values from other social values? I contend that no purely descriptive answers to these questions exist; rather, authors make choices that entail ethical or metaethical assumptions.

Many authors explicitly reflect on their concept of morality. Patricia Churchland, for instance, writes that:

I generally shy away from trying to cobble together a precise definition of ‘moral’ preferring to acknowledge that there is a spectrum of social behaviors, some of which involve matters of great seriousness, and tend to be called moral, such as enslaving captured prisoners or neglecting children, while others involve matters of more minor moment, such as conventions for behavior at a wedding. The boundaries of the concept ‘moral,’ like the boundaries of ‘house’ or ‘vegetable’ are fuzzy. (Churchland, 2011, pp. 9–10)

According to Churchland, morality is about “serious” prosocial behaviors, and the neural basis for this kind of morality developed through a restructuring of the mammalian brain. Central to this restructuring was the chemical oxytocin, a hormone secreted during breastfeeding that performs a bonding function. Churchland locates the historical origin of morality at the point where individual organisms no longer aim solely to ensure their own survival, but begin to care about others’ wellbeing: “At the root of human moral practices are the social desires; most fundamentally, these involve attachment to family members, care for friends, the need to belong” (Churchland, 2011, p. 12).

Like Churchland, most authors seeking to describe “the root of moral practices” or “the ultimate source of ought” seem to view this as a causal-historical occurrence. Typically, when authors use such formulations they are referring to what they think is a particularly relevant causal event in the complex evolution of moral behavior – one that resulted in the development of an ability constitutive of our being moral agents. It is interesting to note that different authors identify different causal occurrences as the first or most important step in the causal chain. As I argue in the following sections, such claims reflect the author’s normative commitment in the form of a mixed claim. The commitment might be fully intentional, a mere coincidence, or somewhere in between. Churchland focuses not on all prosocial behaviors, but specifically on caring behavior as the most important ability in the evolution of morality. Many behavioral scientists, however, focus on a broader range of altruistic behaviors, including cooperation and punishment behaviors that seem to establish norms of fairness or justice (Burkart et al., 2018; Curry, 2016; de Waal, 2006).

Other authors disagree that natural caring behavior, altruistic tendencies, cooperation, or punishment are central to human morality. For instance, Kitcher argues that altruism cannot maintain peaceful cooperation in larger groups, as it tends toward partiality, placing a strong emphasis on the interests of one’s nearest and dearest. While altruism certainly is a relevant precursor to morality, morality in a narrower sense begins as a practice designed to correct *failures* of altruism; this, according to Kitcher, is its primary and most important function (Kitcher, 2011). The disagreement between authors such as Churchland and Kitcher does not concern what happened in evolutionary history—it concerns the metaethical question of what counts as genuinely *moral* behavior. While Churchland focuses on caring behavior as the most central ability for making us moral creatures, many philosophers require that moral behaviors be motivated by the right kinds of reasons (Smith, 1995).

A different group of authors think that emotions are important to the evolution of morality, and perhaps even constitutive of morality itself. Straightforward sentimentalist accounts assume that humans respond to morally relevant scenarios with emotional reactions such as anger, guilt, shame, or contempt. They hold that these scenarios have no common factors by which we may identify them as right or wrong except for the human disposition to respond with sentiments of approval or disapproval (Prinz, 2007). A sentimentalist concept of morality thus assumes that emotions motivate us to act morally. Few have defended full-blooded sentimentalism. Moderate versions hold that morality arises from a combination of emotional abilities and minimal cognitive abilities (Nichols, 2004). Relatedly, some authors label themselves pluralists and defend a broad concept of morality, according to which emotions, norms, and reasoning comprise three equally relevant cores of morality (Kumar & Campbell, 2022). Crucially, each account must decide which emotions are moral emotions. I argue in the next section that this decision contains ethical assumptions.

Some behavioral scientists, as well as some naturalist philosophers, think that the evolutionary function of morality is to enable cooperation among highly interdependent creatures (Smyth, 2017). For instance, in *A Natural History of Morality* Tomasello suggests that human cooperation could have resulted in moral behavior when humans developed a rational attitude toward each other as equally deserving creatures:

The cooperative rationality we are positing here is the ultimate source of the human sense of ‘ought.’ Early human cooperative rationality expands human pro-attitudes to include the welfare of others, it presupposes second-personal agents who consider one another as equally deserving of respect and resources. (Tomasello, 2016, p. 82)

Tomasello seeks evidence for when and how certain social cognitive abilities—such as theory of mind, shared intentionality, and the ability to form joint commitments—evolved. This represents yet another focus on different abilities, distinct from the prosocial behaviors and emotions discussed earlier. According to Tomasello, these abilities, which we share with other primates, are biological precursors to morality. However, what is specific to human morality—and what Tomasello describes as ‘the ultimate source of ought’—is stable cooperation guided by collectively accepted norms, a result of cultural evolution. Within this cooperative framework, we choose trustworthy partners, perform according to role-ideals, respond to requests for help, share resources, or exclude free-riders. Decisions for action can be made either strategically—considering one’s own interests—or morally, meaning “taking into account the interests of a partner of equal deservingness” (Tomasello, 2016, 83). This ideal of treating cooperative partners as equally deserving, according to Tomasello, explains how early hunter-gatherers overcame the hierarchical group structures still seen in chimpanzees, enabling early humans to organize in egalitarian ways. The claim that “cooperative rationality… is the ultimate source of ought” (Tomasello, 2016, 82) is a mixed claim. It identifies a key step in cultural evolution—highlighted as the most relevant in the development of human morality—as the shift toward viewing each other as equally deserving. The implicit claim is that the ideal of equal rights, rather than, for example, caring for others’ well-being, is the most important value to be established in making us moral.

There are modular accounts on the market that assume, contrary to Tomasello, that moral cognition—at least in its rather intuitive and automated form—largely results from biological evolution (for a review, see Sinnott-Armstrong & Miller, 2007). This idea has its origin in Leda Cosmides’ theory of an evolved cheater-detection mechanism, which has the adaptive function of detecting a specific kind of moral violation: intentional cheating in a situation of social exchange. Jonathan Haidt claims that there is an evolved moral faculty that is made up of five evolutionarily acquired intuitions: (1) concern for others, (2) reciprocity and fairness, (3) loyalty to the group, (4) respect for authority and hierarchies, and (5) purity and chastity. On this view, the “moral faculty” comprises a bundle of moral intuitions, which can be further analyzed as an evolved evaluation combined with a feeling of rightness (Clavien & FitzGerald, 2017).

I have already mentioned that several authors, particularly philosophers, identify moral motivation with responsiveness to reasons, and thereby take practical reasoning to be the ultimate moral ability. Richard Joyce (Joyce, 2006) is among the few authors to present an evolutionary account that focuses on practical reasoning. But while there is some indirect evidence regarding the evolution of prosocial behaviors, there is hardly any evidence about the evolution of practical reasoning (Machery & Mallon, 2010).

The foregoing broad overview reveals considerable disunity in the study of the evolution of morality. First, different authors consider different abilities to be moral, including prosocial behaviors, emotions, intuitions, and judgments. Accordingly, each account’s evolutionary narrative highlights certain behavioral or cognitive abilities and leaves out others, usually without justification. Second, authors carve up the moral domain differently by focusing on different values or disvalues, such as harm, wellbeing, fairness, equality, chastity, loyalty, authority, respect, or trust. They choose which among these are social or conventional and which are moral, and these choices are also typically implicit or unexplained. Furthermore, authors tend to distinguish moral norms from other norms by reference to criteria such as universality, categoricity, objectivity, seriousness, or inescapability.

Finally, while it is possible to defend a monist position, according to which morality must have emerged from the development of a single ability, there is a strong pluralistic tendency in the literature, albeit with different variants of pluralism. 1) Moderate pluralism: for example Tomasello claims that morality evolved in several steps, but he still highlights one step as the most relevant. 2) natural kind pluralism: in their recent book by Kumar and Campbell develops a natural kind pluralist position, arguing that different abilities (prosocial behaviors, emotions, and cognitive abilities) contributed equally to the evolution of morality. However, they still treat morality as a natural kind, which distinguishes them from 3) pragmatic pluralism: authors who adopt pragmatic pluralism view the concept of morality not as a joint-carving concept, but as one chosen with regard to context-sensitive interests (Ludwig & Ruphy, 2021; Westra & Andrews, 2022).

In summary, there is extensive disunity between different concepts of morality and broader understandings of the main causal factors that make us moral. One might think that this problem is superficial. After all, can we not distinguish a broad, descriptive notion of morality that includes prosocial behaviors and emotions from a narrow notion consisting of judgments that highlight particular formal features? Can we not simply settle on one proper a priori criterion from philosophy? Why not search for a proper descriptive concept of morality in moral psychology? Is the tendency toward pluralism in recent literature not a sign that authors are giving up their implicit normative commitments anyhow? I will argue that none of these suggestions is a viable option in the next section.

## The search for a descriptive concept

In the previous section, I identified a considerable disunity among concepts of morality in a narrower sense and within the general understanding of the major causal factors involved in our becoming moral creatures. Authors disagree about what counts as moral abilities, moral norms and values, and formal criteria for morality. In this section, I discuss whether there might nevertheless be an uncontroversial conceptual criterion for favoring one concept of morality over another, by looking at each dimension of disagreement in turn. I then review suggestions from moral psychology to assess whether we can define a descriptive concept on empirical grounds alone. The range of ethical and metaethical claims involved in mixed claims about the evolution of morality will be made explicit along the way.

### Moral abilities

One source of disunity is that authors consider different abilities to be constitutive of, or particularly causally relevant for, our status as moral creatures. These abilities range from the ability to show prosocial behaviors to the ability to show moral emotions, modular cognitive processes, or practical reasoning. How can we decide whether one such ability is more relevant than the others?

According to some accounts, the evolution of emotions such as guilt and resentment has been the most relevant constitutive step in the evolution of morality (Prinz, 2007); according to others, it is one of the more relevant steps among others (Kumar & Campbell, 2022; Nichols, 2004). Still others focus on one particular moral emotion such as guilt (Joyce, 2006); others hardly mention emotions at all (Tomasello, 2016). The claim that emotions are in some sense constitutive of our ability to be moral implies a commitment to some form of sentimentalism and is therefore a controversial metaethical claim. However, the same should be said of views that assume that emotions are *not* part of the core abilities that make us moral. It is not possible to proceed in this debate without committing to *some* controversial metaethical claim about what constitutes morality.

Furthermore, different accounts consider different emotions to be more or less morally relevant. Joyce takes guilt to be a central element in the evolution of consciousness (Joyce, 2006). Shame could be considered equally relevant, though, because it is also a self-conscious emotion that responds to norm-violations and Jesse Prinz in fact does think of guilt and shame being equally relevant for the evolution of morality (Prinz, 2007). There is no obvious answer in the evolution of emotions. But there is a difference in how guilt and shame have been evaluated during the past few centuries in Europe. The historian Ute Frevert, for instance, analyzes the decline of public shaming in the context of the European Enlightenment; “In highly individualistic societies that put personal autonomy first and community values second, public shaming seems no longer acceptable” (Frevert, 2016, p. 59). In contemporary Western societies guilt tends to be considered an emotion that motivates autonomous individuals to rationally assess whether they have lived up to moral standards. In contrast, shame is seen as an emotion that can be (ab)used to enforce social norms by threatening individuals with public humiliation if they violate certain rules. According to this view shame motivates norm conformity only to the extent that people wish to avoid being shamed, while guilt is a response of an autonomous individual. Authors like Prinz argue that public shaming is integral to the norm-enforcing practices involved in the cultural evolution of morality. In contrast, authors like Joyce seem to implicitly carve up the concept of morality according to the standards familiar from Kant and the European enlightenment. In both cases relevant normative background assumptions influence whether shame is considered as a moral emotion.

This argument generalizes: I am not aware of any criterion that can carve emotions into moral and nonmoral ones without referring to further moral values, norms, or metaethical criteria. If we must refer to any of these to explain which emotions are relevant for our ability to be moral, then it seems that these values rather than emotions should be our primary focus. Thus, pointing to core abilities that play a constitutive role in the evolution of our morality can hardly be done without committing oneself to controversial metaethical position like sentimentalism. Furthermore, even fine-grained differences such as whether shame is considered a moral emotion or not are tied to normative background assumptions e.g. the acceptance of enlightenment ideals about autonomous individuals and the resulting distinction between guilt as a moral and shame as a social emotion. One might nevertheless think that we deliver an adequate concept of morality on a priori grounds. I discuss this option in the next section.

### The moral–conventional distinction

Morality is generally taken to refer to a set of norms and principles that govern our actions with respect to one another (Driver, 2022). Moral norms allow us to determine which actions are good or bad, obligatory, permitted, forbidden, virtuous, or vicious. However, a more controversial question is how to distinguish moral norms from other types of norms, such as conventional norms like those of etiquette. There is a considerable literature in philosophy and psychology dedicated to making such a distinction (Brennan et al., 2013). We can roughly divide the positions within this literature into those with content- or value-oriented criteria and those with formal criteria. Formal criteria refer to the formal properties of principles recalled in moral judgments, such as their scope or conditions of application, or the nature of normativity.

In the previous section, we saw that harm, wellbeing, fairness, equality, chastity, loyalty, authority, respect, and trust are among the values to be found in the literature, and that many authors pick out only a subset as morally relevant while others suggest a broader scope. What is the criterion that delineates moral values moral from others? The history of ethics contains several accounts that treat one particular value as the most relevant ethical one. According to a Hobbesian account, for instance, harm-avoidance is the most central moral value within a society. In contrast, Utilitarian morality consists in maximizing the pleasure or happiness of the greatest number. A Kantian account suggests that equal rights and autonomy are the most important values, whereas a Rawlsian account might favor fairness or justice. A claim about e.g. “the ultimate source of ought” that mainly refers to the evolution of equality-norms appears to be a mixed claim, insofar as it leans more toward a Kantian approach than towards any other.

Formal criteria aim to define morality using principles recalled in moral judgments, such as their scope, conditions of application, or particular nature of normativity. Some highly prominent criteria include universalizability, which is the idea that moral rules are characterized by the fact that they hold for everyone, everywhere, at all times. Closely related is the view that we think of moral norms as objective, categorical, or unconditional. According to these criteria, moral judgments evaluate actions independently of the agent’s interests and purposes. This distinguishes them from instrumental judgments, saying that in order to achieve purpose Z, action H should be performed. Another criterion concerns overridingness: moral obligations are necessarily more binding and obligatory than other evaluations (Hare, 1952). Authors such as Hare sought a descriptive concept of morality that could be picked out by a criterion, or by several criteria, that provided necessary and sufficient conditions.

The most apparent problem with formal criteria is the sheer number of them on the market (see Driver, 2022 and Machery & Stich, 2022, for more detailed overviews). The purpose of this paper is not to advocate for or against any criterion; instead, it suffices to examine what makes a criterion controversial. Universalizability for example is by many considered a criterion that is insufficient for morality. Consider an English colonist who, though thousands of miles away from England, can distinguish “the gentleman” from “the savage” based on a lapse in dress style: he appears to be universalizing conventions to justify classism (Southwood, 2011). Similarly, many normative judgments concern social conventions that Kant would deem categorical. The directive, for example, that one should hold a fork in one’s left hand hardly seems contingent on viewing this task as an individual goal or end (Foot, 1972).

Nor are formal criteria necessary. Consider Hare’s criterion of overridingness and Churchland’s comparable claim that we regard moral norm violations as especially serious and severe. A white lie, or stealing potatoes when one is starving, strike us as moral transgressions that are subordinate to pragmatic goals or social norms, or even to legal rules. As we shall see, a descriptive definition of morality need not offer exceptionless necessary and sufficient criteria. Instead, we can think of a descriptive concept as picking out a natural kind, and we can think of a natural kind as a nomological cluster (Machery & Stich, 2022).

But the lack of a formal criterion that provides necessary and sufficient conditions does not help to settle philosophical disagreements about the different candidates on the market. To complicate matters, some philosophers criticize the focus on formal criteria as a misguided search for a moral system (Williams, 1985). In their view, it is not clear that morality can be captured with a formal criterion. For instance, virtue ethicists worry that the use of formal criteria to distinguish between moral and conventional norms will render the former more binding than the latter, but whether this is true should be decided within a concrete context (Baier, 1985).

For our purposes, it suffices to acknowledge the absence of a philosophical consensus on a formal criterion for moral versus conventional norms that does not make controversial normative assumptions. Philosophers’ failure to discover an uncontroversial definition of morality does not preclude the possibility that an empirical search for a natural kind might offer a joint carving descriptive concept. Whether claims about the origin of morality are considered mixed claims depends at least partly on whether any joint-carving descriptive concept is available. The next section will examine the empirical debate about the existence of such a natural psychological kind.

### The limitations of joint-carving in psychology

The question of how best to distinguish moral from conventional norms is important not only to philosophy but also to psychology as well. Beginning in the 1970s, the developmental psychologist Elliott Turiel and his colleagues developed the most prominent proposal for distinguishing the moral from the conventional, which shaped the debate in anthropology and philosophy for decades (Haidt et al., 1993; Machery & Stich, 2022; Nichols, 2004; Nucci & Turiel, 1978; Turiel, 1983).

Turiel’s main hypothesis is that young children can distinguish moral and conventional norms along several dimensions, and that this ability points to a division of human normative cognition into cognition about social and moral norms that arises early in development (Nucci & Turiel, 1978, p. 401; Turiel, 1983). Turiel and colleagues devised an empirical test showing systematic differences in children’s judgments about moral versus conventional transgressions. A well-known finding of these studies suggests that children as young as three or four years old can make such a distinction. Turiel concludes that, from an early age, we treat moral norms as universalizable and independent of authorities, and see particularly moral norms transgressed in cases of harm, unfairness, and violations of equal rights. By contrast, we consider conventions to be local norms, resulting from conventions or authorities, that concern etiquette and other social arrangements.

In principle, this is precisely the kind of evidence required to carve up a descriptive concept and a descriptive concept is one that is free of normative commitments to particular ethical theories. If it turns out that humans as young as three or four cross-culturally distinguish moral from conventional norms along the above criteria, then strong evidence exists of a psychological core competence with roots in evolutionary history. At first sight, the explanandum appears to be founded on an empirically grounded observation of the phenomenon in question. It thus appears to be pre-theoretical: that the formal and content-oriented criteria can be found in developmentally early behavior justifies narrowing the moral domain. We seem to be in a good position to offer a concept of a complex, but stable, human ability by focusing on these criteria—if we refrain from sneaking in ethical or metaethical beliefs. The children’s responses allow us to infer an underlying cognitive ability, from a stable means of distinguishing between moral and conventional norm violations, that can be explored across disciplines. We thus have a promising candidate for a purely descriptive concept that is still sufficiently concise to underlie an evolutionary narrative.

There is an emerging consensus, however, that Turiel’s understanding of morality relies on value-laden assumptions, and that it is far from empirically uncontroversial. The studies and their implications have been discussed in detail elsewhere (Machery & Stich, 2022; O’Neill & Machery, 2019). For our purposes, Haidt makes the important objection that Turiel’s concept of morality “does not travel well” (Haidt, 2012, p. 22). Haidt argues that, across cultures, values such as authority, loyalty, and chastity are frequently treated as universalizable and authority-independent norms, while Turiel thought of them as conventional ones. The narrow focus on fairness and harm norms as the core moral domain thus stemmed from Western psychologists’ conceptual bias toward democratic, liberal concepts of morality.

Similarly, comparative anthropologists have argued that many cultures treat loyalty and authority as moral values insofar as they serve group cohesion, and that many religious contexts treat purity and chastity as universalizable and especially serious. In health contexts, moreover, we often treat norms associated with disgust as moral norms (Nichols, 2004; Shweder et al., 1997). These empirical findings reinforce the suspicion that an understanding of morality focusing on values, if it is to be understood as a descriptive project, is question-begging: it entails beliefs about which values should count as moral in the first place.

In response, some authors have suggested that morality, in a narrow sense, most likely does not result from biological evolution (Machery & Mallon, 2010); that it does not pick out a natural kind (Stich, 2019); and that it might prove more fruitful to look for basic normative abilities found in infants and other species (Andrews, 2020; O’Neill, 2017; O’Neill & Machery, 2019; Westra & Andrews, 2022). This line of reasoning is strengthened by similar worries, raised by philosophers and neuroscientists, about the disunity of moral judgments and their locations in the brain (Sinnott-Armstrong & Wheatley, 2014; Young & Dungan, 2012). In a recent review, Machery and Stich concluded that it is still an empirically open question whether there is a natural kind that can be picked out by a descriptive concept (Machery & Stich, 2022). Other authors defend a pluralist natural kind view, significantly broadening what they take to fall under the descriptive concept of morality while still defending a distinction between moral and conventional norms (Kumar & Campbell, 2022; Reichlin, 2024). Still others approach the subject as pragmatic pluralists, contending that there are no joints in nature and therefore scientists are picking a concept of morality according to their pragmatic interests.

I am happy to accept the possibility that future research might deliver a joint-carving concept of morality. Currently, however, there is no uncontroversial descriptive concept available. In the next section, I examine how moderate pluralists and natural kind pluralists, despite having a broad concept of morality in mind, are still making mixed claims.

## Analyzing mixed claims

Authors who have written on the evolution of morality entertain a broad variety of ideas about what morality is and what the crucial steps in its evolution are. There are no uncontroversial grounds, a priori or empirical, that would allow them to articulate a descriptive concept of morality that does not collapse into a concept of social norms.^4^ In the following, I first demonstrate how, as a result, the literature on the evolution of morality is prone to mixed claims; I discuss some typical examples and I argue that not making mixed claims explicit often leads to biased narratives or fallacious reasoning. In the next section, I discuss how research could benefit from making mixed claims explicit.

I have already demonstrated that a typical move in the literature is to point to a causal-historical occurrence e.g. as “the ultimate source of ought” or “the first sparks of morality.” I suggest, as a hermeneutical rule of thumb, that these formulations will be the go-to place to find implicit normative assumptions. Consequently, wherever authors most emphatically talk about “the true origin of morality” or “the earliest ethical code”, we can treat their putative causal-historical claims as mixed claims and raise questions about what their mixedness means with regard to empirical plausibility on the one hand and conceptual ethics on the other.^5^

According to Churchland, the evolution of morality can be traced back to that of the mammalian brain: “At the root of human moral practices are the social desires; most fundamentally, these involve attachment to family members, care for friends, the need to belong” (Churchland, 2011, p. 12). Many philosophers will find this focus misguided and claim that we should instead focus on the human ability to be motivated by reasons or to form moral judgments in reasoning about abstract principles. Accordingly, it has been suggested that a significant portion of the debate concerns precursors of morality, such as empathy, and not morality as such—simply because there is very little empirical evidence about the evolution of higher cognitive abilities (Machery & Mallon, 2010). This observation certainly contains some truth, and it explains, at least in part, an eye-catching imbalance in the literature between the empirical focus on prosocial behaviors and the philosophical focus on moral judgments and normative reasons. Researchers focus on prosocial behaviors for pragmatic reasons: there is evidence to look at.

However, even if an author such as Churchland focuses on the ability to care for others for prudential reasons alone, the focus still contains a normative dimension that warrants further exploration: Carol Gilligan, one of the most prominent advocates of care ethics, argues that developmental psychologists such as Lawrence Kohlberg have mistakenly characterized a “morality of rights” and independence from others as superior to, rather than merely different from, a “morality of care” which emphasizes empathy, perspective-taking, and the ability to form relationships and care for others wellbeing (Gilligan, 1982). While Churchland neither quotes Gilligan nor explicitly references care ethics, her emphasis on fundamental behavioral and emotional capacities—tracing back to the evolution of mammals and the ability of mothers to care for others’ wellbeing—aligns closely with this tradition. Even if a particular choice is made for purely pragmatic reasons, it still leads to a narrative that emphasizes certain ethical assumptions over others. For instance, the view that we become moral when we begin to care for others is biased in that it elevates caring for others’ wellbeing as the ultimate moral virtue.

A different understanding of what makes us moral, along with a correspondingly different narrative, can be found in the works of Kitcher, who locates the primary function of ethics in the regulation of altruistic behavior (Kitcher, 2011) As noted, part of Kitcher’s departure from Churchland stems from a metaethical claim about what constitutes genuine moral motivation—specifically, that moral behavior must be motivated by reason. Kitcher interprets “the earliest ethical code” as emerging from “discussions around the camp-fire,” and argues that “Equality, even a commitment to egalitarianism, was important in the earliest phases of the ethical project”. (Kitcher, 2011, p. 96). The historical evidence, however, that Kitcher cites in support of this claim is controversial (e.g., Graeber & Wengrow, 2021).

Interpreting the campfire discussions of early hunter-gatherers as “the earliest phase of the ethical project” is another biased narrative that results from a different understanding of what makes us moral. Just as Churchland emphasizes the caring relationship and the wellbeing of others, Kitcher’s approach focuses on the recognition of equal rights. When comparing their accounts, there is little direct disagreement about empirical facts—Kitcher does not doubt that the ability to care for others evolved in the way that Churchland describes. But he takes it to be a precursor to genuine morality that evolved much later. The difference between the two accounts is metaethical in nature. For Kitcher, moral behaviors must be motivated by the right kinds of reasons. The claim that fixing the failure of altruism is the first function of the ethical project, as well as the claim that egalitarian discussions among hunter-gatherers were “the earliest phase of the ethical project,” are clear cases of mixed claims grounded in an egalitarian understanding of morality.

In fact, egalitarian beliefs play a prominent role in current research. Tomasello traces the origins of morality to an early form of cooperative rationality that expanded human pro-social attitudes to include concerns for others’ welfare. This view assumes second-personal agents who consider one another as equally deserving of respect and resources (Tomasello, 2016, p. 82). Tomasello borrows the Kantian notion of agents who view each other as ends in themselves from Darwall (Darwall, 2006; Roughley, 2018). In their recent book, Kumar and Campbell argue that emotions of trust and respect are psychological mechanisms that enabled our ancestors to cooperate and the create egalitarian groups. With this and other claims Kumar and Campbell broaden the concept of morality and the scope of psychological mechanisms involved in making us moral creatures (Kumar & Campbell, 2022). At first glance, it might seem that the scope of what the authors call ‘core moral abilities’ is so broad that it may collapse into normative abilities. On a closer look, however, we see that this is not the case. Rather, something crucial for current research about the evolution of morality becomes visible: empirical arguments and normative beliefs are hard to disentangle. Kumar and Campbell’s taxonomy of moral norms, for example, departs from Haidt’s insofar as the former argue that core moral norms exclude norms of purity and authority. According to Kumar and Campbell, this is because purity and authority norms became relevant only during the past few thousand years with the development of large-scale societies. They do not doubt, however, that the emotions of disgust, pride, and shame—commonly associated with norms of purity and authority—were already present in the early days of *Homo sapiens*. But disgust had not yet been co-opted for purity norms, and authority “had not yet been elevated to the status of a norm” (Kumar & Campbell, 2022, p. 95). These are causal-historical claims made about complex social events in the past, making them fairly hard to test; I would be interested to see a discussion of the kinds of evidence that could bolster or falsify the authors’ claims. At the same time, excluding purity and authority norms from the “core of moral norms” does well to set the focus on norms that facilitate egalitarian forms of cooperation, while leaving out those that function primarily in hierarchically structured groups. It is hard to avoid the impression that the resulting moral taxonomy is empirically underdetermined while conveniently carving moral and non-moral emotions along egalitarian lines.

This is particularly interesting because Kumar and Campbell defend a natural kind pluralism and one might think that their account in broadening the concept of morality comes close to a descriptive concept of morality free of any ethical or metaethical commitments. But this is deceiving. First of all, the idea that behavior, emotions and reasoning all play a role in the constitution of morality is itself a metaethical view. More relevant here is the fact that authors mix assumptions about which values should be considered moral with descriptive claims about which norms became relevant at different stages of evolution. The bottom line is that, where natural kind pluralism about morality does not collapse into a broader concept of normativity, what counts as moral and what doesn’t should be clearly defined with reference to explicitly normative criteria. It is problematic when empirically underdetermined claims take the place of what should be explicitly recognized as a mixed claim.

Empirically underdetermined claims are fairly common in research about the evolution of social and cognitive abilities.^6^ I shall argue below that their role in shaping narratives need not be problematic as long as possible biases, value-assumptions etc. are made explicit. A major problem apart from biased narratives is that mixed claims taken as descriptive claims can be mistaken with regard to their justificatory powers. Kitcher proposes a metaethical approach according to which ethical statements’ truth can be grounded in ethical codes’ functionality (see Müller, 2020, for a more detailed analysis). Whether moral change is progressive or not can be answered by checking whether a rule introduced to the ethical code would actually remedy the failures of altruism. It seems as if Kitcher wants to suggest that remedying altruism’s failures is the relevant function of ethics because it is its *original* function (Kitcher, 2011, pp. 221, 225; Müller, 2020). However, the claim that avoiding altruism’s failures by developing ethical codes around the camp-fire is “the original function of ethics” is a mixed claim in the first place. It entails a descriptive hypothesis that a certain kind of ethical bargaining took place in a certain form at a certain time, and it entails the normative background assumption that being able to collectively fix altruism failures in an egalitarian setting is the core constituent of ethical practice. Understood this way, Kitcher’s grounding of metaethical truth begins to appear circular. When authors are not explicit about the fact that they are making mixed claims rather than merely stating historical facts, they risk drawing fallacious inferences. The lack of transparency about normative presuppositions can obscure what can reasonably be inferred from a certain claim.

Even clearly descriptive accounts that do not aim to derive something like a notion of moral progress out of an evolutionary narrative, however, entail ethical or metaethical background-assumptions. Tomasello’s description of the evolution of human cooperation as “social selection against bullies” (Tomasello, 2016, p. 43) is a prime example of a descriptive account claiming that humans are distinguished from their ancestors because humans developed the ability to treat each other as equals. The empirical findings that Tomasello presents about bullying among chimpanzees and cooperation among children are undoubtedly fascinating. However, it is difficult to interpret the claim that treating each other as equals is the ultimate source of ought as anything other than a mixed claim, entailing a normative commitment to egalitarianism. If this is not made explicit, it biases the resulting evolutionary narrative.

We have seen how outright fallacious reasoning and biased narratives result from implicit mixed claims that are left unpacked. In both cases, research would benefit from disentangling the ethical and metaethical assumptions embedded in these claims. In the next section, I address concerns that embracing mixed claims might compromise the objectivity of research on the evolution of morality. I then outline a new vision for the field that appropriately incorporates ethical and metaethical assumptions and explicitly negotiates them.

## Negotiating what makes us moral

I have argued that the scientific research about the evolution of morality involves mixed claims, as morality is not a joint-carving concept, and empirical evidence about the evolution of social and cognitive abilities is often underdetermined. Consequently, claims about the origin of morality inevitably entail ethical or metaethical assumptions. I have demonstrated the range of metaethical and ethical implications arising from these claims and shown how the epistemic aims of transparency and comparability are undermined by the close entanglement of empirically underdetermined claims and normative implications. As a result, some accounts paint biased pictures about our moral nature, while others fall into drawing circular conclusions about the nature of moral progress.

What would a science of the evolution of morality look like, if it properly acknowledged both empirical claims and ethical or metaethical assumptions? According to the value-free understanding of objectivity, values should not determine or influence the acceptance of a hypothesis (Douglas, 2009). As we have seen, however, values do in fact influence the acceptance of mixed claims, as the initial value judgment precludes certain findings. For instance, different theories of wellbeing differently constrain the range of available findings.

Alexandrova suggests a form of procedural objectivity that focuses on how the relevant values are handled. Procedural objectivity emphasizes the transparency, legitimacy, and resistance of the inquiry process to manipulation by specific individuals or groups. When applied to values, the first is to make the underlying value assumptions explicit— unearthing the values that the methods and measures presuppose. In some cases a single sentence in an article’s methods section may suffice. However, in the study of the evolution of morality, authors often construct complex narratives that blend empirically underdetermined claims with assumptions about what morality is and which causal steps in its development were most relevant. What matters most is to be more explicit in acknowledging alternative presuppositions. Authors should invest time and effort discussing how their accounts relate to others. They should not reduce these comparisons to empirical differences; instead, they should explore possible disagreements about ethical and metaethical assumptions.

A second step in ensuring procedural objectivity is to search for robustness. Measures of wellbeing, for instance, can sometimes be “robust” to fundamental philosophical disagreements. Measures of child wellbeing attempt to capture conditions that, if realized in childhood, enable children to grow up happier, healthier, and more socially connected. These measures hold up across major theories of wellbeing—whether experiential, reflective, or objective. Here, robustness refers to an invariance across different concepts of wellbeing, which grants some mixed claims “objectivity on the cheap” (Alexandrova, 2021, p. 101). In the context of morality, values such as harm-avoidance and fairness are generally uncontroversial across theories (Reichlin, 2024). What is controversial is the hierarchical ranking among these values, and whether the scope of moral values should be broadened to include values such as authority or purity. When authors narrow the scope of values for empirical reasons, they should be transparent about both the status of the evidence and normative discussions surrounding the issue. In other words, authors should at least consider the option that they are making mixed claims.

When robustness cannot be achieved—due to disagreements about the relevant values—Alexandrova proposes a third step toward procedural objectivity: consulting the relevant parties. The science of wellbeing is a diverse field, but much of it serves practical applications, such as medical treatments. In such cases the relevant parties are the stakeholders for whom the research results matter. In contrast, accounts of the evolution of morality do not seem to have specific stakeholder groups; insofar as they develop historical narratives, one can doubt whether they have practical applications at all—at least it is more difficult to directly see what the practical implications are. However, because they develop theories about the evolution of *morality*, they concern each and every human being alike. Values in mixed claims about the evolution of morality can thus be considered parts of the science of human nature (Hannon & Lewens, 2018; Kronfeldner, 2018), whose normative implications have been widely discussed. The debate about the evolution of morality differs from, say, the debate about the evolution of bone structures because scientists, philosophers, and the broader public perceive the former as relevant to how we live together as moral creatures. As Kumar and Campbell state in the preface of their recent book, “Morality isn’t simply one facet of human nature. It’s why humanity grew out of nature” (Kumar & Campbell, 2022, p. xi). It is enlightening to view the conceptual disunity about morality as an implicit negotiation about what exactly caused us to “grow out of nature.” Unsurprisingly, egalitarian authors emphasize the abilities that enable humans to treat each other as equals often showing less interest in emotions linked to hierarchical organization. This focus is fine as long as it is explicit. However, when a book is titled *A Natural History of Morality* (Tomasello, 2016), a narrow focus on values particularly relevant to the Western tradition—if not explicitly acknowledged—seems biased. 

Christopher Boehm’s book *Hierarchies in the Forest*, frequently cited by authors with egalitarian beliefs, makes the author’s value-commitments explicit. Boehm argues that, while chimpanzees have stable hierarchies, early hunter-gatherers were able to organize themselves into relatively egalitarian groups. The book opens as follows:

To one who is living one’s life as a democrat, egalitarianism is a topic that affects not only the head, but the heart. My heart was in this book, not only in the research that underlies it, but in its writing.... It is this essential political leverage in the political decision-making process that keeps alive human freedom and human rights as we know them, and I shall argue that this type of political stance is quite ancient. (Boehm, 2001, p. vii)

Does the disclaimer, that the book will argue that the author’s favored political stance is “quite ancient,” put its scientific legitimacy into question? I think not. If the author were to make egalitarianism seem “more ancient” than it actually is—perhaps to lend it more gravitas—then that would provide good reason to question its scientific legitimacy. In contrast an explicit disclaimer about the author’s own normative beliefs seems to invite critical questions from the audience: “Is it really that old, though?” “Is the ancient behavior you describe really egalitarian?” “Isn’t dominant behavior to establish hierarchies even older?” This is the way we should approach research that entails both underdetermined evidence and mixed claims.

Finally, Alexandrova holds not only that we cannot get rid of mixed claims but also that we *should not* try to do so. The main reason to aim for a separation of descriptive scientific claims from normative ones is the value-free ideal that comes with a propagated division of labor—between scientists making descriptive claims on the one hand, and ethicists making normative ones on the other. We have already seen that the objectivity of science does not require this distinction; being explicit about values does a much better job here. Moreover, Alexandrova argues that such a division of labor also ignores or devalues scientists’ knowledge about values—knowledge that they acquired by studying the facts. Because of their expertise, scientists are in a better position to make certain normative choices: “It is because developmental psychologists know the effect of, say, institutionalisation of orphans that they believe attachment to be crucial to child well-being” (Alexandrova, 2021, p. 92). Of course, the idea is not that scientific expertise renders ethical considerations redundant, but rather that certain normative considerations benefit from, and are difficult to disentangle from, scientific expertise.

This holds true for the research on the evolution of morality as well. Thus, being explicit about mixed claims within the field will not diminish its relevance. Rather, it will help the field to avoid biases and fallacies, to unpack the normative knowledge shared by behavioral scientists and comparative psychologists and give this knowledge a proper place in research. Consider, for instance, Tomasello’s description of parts of the evolution of cooperation as a “social selection against bullies” (Tomasello, 2016, p. 43) which enabled humans to live in large groups more peacefully than chimpanzees ever could. The ultimate source of ought, according to this view, is the ability to see each other as equally deserving. Recognizing this as an explicitly mixed claim—and unpacking egalitarianian background assumptions—would open up an entirely new dimension of the book. When Tomasello tells us about the abilities that help to stabilize cooperation in infants, and how these abilities enable joint commitments of which chimpanzees are not capable, we could take this to be a partly normative claim about the abilities that we need to strengthen during infancy *if we want* children to learn how to live in egalitarian societies. We would learn not only what differentiates us from chimps and what this difference might tell us about our evolution: we would also learn *why* Tomasello takes this difference to be of core relevance to human wellbeing and peaceful cooperation. The book would not only tell us about the most likely historical origins of cooperation but also investigate these hypothetical origins to investigate the conditions under which we can cooperate peacefully, what our most likely problems are, and what promising strategies exist to handle them. To think of claims about the evolution of morality as mixed claims from the start would lead not only to greater transparency and comparability—it would make it possible to unpack the very normative knowledge that behavioral scientists and comparative psychologists accumulate throughout their research.

## Conclusion

I have argued in this paper that there is no way to carve up what morality is in a way that is both purely descriptive and sufficiently concise to inform an evolutionary narrative. As a result, claims about “what truly makes us moral” are never mere causal-historical claims. Instead, they are mixed claims that involve ethical and metaethical implications. They combine a causal-historical hypothesis with assumptions about what the relevant abilities or norms that make us moral are. The debate would benefit significantly from making this typically implicit normative dimension explicit. It would further the epistemic aims of transparency and comparability. It would avoid biases and fallacious reasoning regarding the nature of moral progress. Finally, it would help to unpack the normative knowledge that behavioral scientists and comparative psychologists accumulate, and it would put us in a better position to negotiate, in light of the historical evidence, what makes us moral creatures.

## Data Availability

N/A.

## Footnotes

1 One could also think of wellbeing and related concepts as “thick concepts”: concepts with both evaluative and descriptive content (Kirchin, 2013). Alexandrova does not do this, because there are so many foundational controversies about the nature of thick concepts (do they undermine the possibility of a moral theory? Do they expose the limits of the fact/value distinction, etc.) that she wishes to avoid. A further reason to speak in terms of mixed claims is that these apply to a broader range of cases than concepts alone: we shall see that some authors might largely agree on a concept of morality but differ in the mixed claims they make about the “ultimate source of ought” and so on.

2 Alexandrova speaks about different *conceptions* of wellbeing. The difference between a concept and a conception is famously introduced by Rawls to make sense of the idea that different theorists can share the concept of, for example, democracy, but still debate different interpretations of it by proposing different conceptions (i.e., different definitions or different background theories) of democracy. Adherence to the conceptual “core” ensures that using the term “democracy” does not cause them to talk past each other and that the proposed conceptions are in fact conceptions of democracy. Although this has the advantage that we can make sense of meaningful disputes where the parties do not talk past each other, it has been objected that there usually is no such “common core” of a concept on which authors could agree (Brun, 2022). This is exactly what I think is true with regard to morality, so I do not use the concept/conception distinction, but rather speak of how the concept is used or understood, or of how the theoretical background assumptions differ.

3 When discussing abilities here, I do not intend to settle on a particular view of what kind of powers abilities are. Specifically, most philosophers hold that abilities are powers that relate an agent to action. This definition inherits the challenges involved in distinguishing between actions and non-actions, where on most accounts, prosocial behaviors would not count as actions. Nevertheless, I use ‘ability’ as an umbrella term because I aim to show that authors who consider prosocial behaviors to be central to a narrative on the evolution of morality make theoretical assumptions on what it means to be a moral agent in doing so.

4 There is a deeper philosophical question at play here: is there something intrinsic to the concept of morality that makes it impossible to provide a descriptive yet concise definition? Does defining morality necessarily require drawing on ethical or metaethical background assumption? I have not addressed this question in this paper; instead I have confined my argument to an inference to the best explanation: thus far, neither philosophical analysis nor empirical psychology has settled on anything like a coherent definition of morality – suggesting that perhaps no such definition exists.

5 In fact, I think that the whole debate about the evolution of morality can be understood, in part, as a metalinguistic negotiation of what we should mean by the concept “morality”; by picking their favorite abilities, values, and formal criteria, authors implicitly show what their favorite criteria are (Plunkett, 2015; Plunkett & Sundell, 2013). I do not develop the idea of a metalinguistic negotiation further in this paper, but simply show what the debate could gain by making this negotiation explicit.

6 A common concern is that these narratives are nothing more than just-so stories. Louise Antony has recently criticized Kumar and Campbell’s book for being just that (Antony, 2023). I leave it for another paper to argue, that while all accounts of the evolution of morality are underdetermined and speculative to some degree, we can draw on Adrian Currie’s distinction between “vicious” and “virtuous” speculation to assess these narratives more fruitfully (Currie, 2023). Mixed claims, however, will tend to fall on the side of vicious speculation, where their normative background assumptions remain unacknowledged and implicit.

## References

[CR1] Alexandrova, A. (2018). Can the science of well-being be objective? *The British Journal for the Philosophy of Science,**69*(2), 421–445. 10.1093/bjps/axw027

[CR2] Alexandrova, A. (2021). *A philosophy for the science of well-being*. Oxford University Press.

[CR3] Antony, L. (2023). *Review of a better Ape: The evolution of the moral mind and how it made us human*, by Victor Kumar and Richmond Campbell. https://ndpr.nd.edu/reviews/abetter-ape-the-evolution-of-the-moral-mind-and-how-it-made-us-human/

[CR4] Andrews, K. (2020). Naive normativity: The social foundation of moral cognition. *The Journal of the American Philosophical Association,**6*(1), 36–56.

[CR5] Baier, A. (1985). *Postures of the mind*. University of Minnesota Press.

[CR6] Boehm, C. (2001). *Hierarchy in the forest: The evolution of Egalitarian behavior*. Harvard University Press.

[CR7] Brennan, G., Eriksson, L., Goodin, R., & Southwood, N. (Eds.). (2013). *Explaining norms*. Oxford University Press.

[CR8] Brun, G. (2022). Re-engineering contested concepts. A reflective-equilibrium approach. *Synthese,**200*(2), 168. 10.1007/s11229-022-03556-7 35509852 10.1007/s11229-022-03556-7 PMC9012703

[CR9] Burkart, J. M., Brügger, R., & van Schaik, C. (2018). Evolutionary origins of morality: Insights from non-human primates. *Frontiers in Sociology,**3*(17), 3–17. 10.3389/fsoc.2018.00017

[CR10] Churchland, P. (2011). *Braintrust: What neuroscience tells us about morality*. First Princeton Science Library paperback edition. Princeton Science Library. Princeton University Press.

[CR11] Clavien, C., & FitzGerald, C. (2017). The evolution of moral intuitions and their feeling of rightness. In Joyce, R. (Ed.), *The Routledge Handbook of Evolution and Philosophy*. Routledge.

[CR12] Currie, A. (2023). Science & Speculation. *Erkenntnis,**88*(2), 597–619. 10.1007/s10670-020-00370-w

[CR13] Curry, O. S. (2016). Morality as cooperation: A problem-centred approach. In Shackelford, T. K., & Hansen, R. D. (Eds.), *The evolution of morality* (pp. 27–51). Evolutionary Psychology. Springer. 10.1007/978-3-319-19671-8_2

[CR14] Curry, O. S., Mullins, D. A., & Whitehouse, H. (2019). Is it good to cooperate? testing the theory of morality-as-cooperation in 60 societies. *Current Anthropology,**60*(1), 47–69. 10.1086/701478

[CR15] Darwall, S. (2006). *The second-person standpoint: Morality, respect, and accountability*. Harvard University Press.

[CR16] de Waal, F. (2006). *Primates and philosophers: How morality evolved. The university center for human values series*. Princeton University Press.

[CR17] Diener, Ed., Lucas, R., Schimmack, U., & Helliwell, J. (Eds.). (2009). *Well-being for public policy. Series in positive psychology*. Oxford University Press.

[CR18] Douglas, H. (2009). *Science, Policy, and the Value-Free Ideal*. University of Pittsburgh Press.

[CR19] Driver, J. (2022). Moral theory. In Zalta, E. (Ed.), *Stanford Encyclopedia of Philosophy*. https://plato.stanford.edu/entries/moral-theory/

[CR20] Foot, P. (1972). Morality as a system of hypothetical imperatives. *Philosophical Review,**81*(3), 305–316.

[CR21] Frevert, U. (2016). The history of emotions. In *Handbook of Emotions* (4th edn, pp. 49–65). Guilford Publications.

[CR22] Gilligan, C. (1982). *In a different voice: Psychological theory and women’s development*. Harvard University Press.

[CR23] Graeber, D., & Wengrow, D. (2021). *The dawn of everything: A new history of humanity*. Farrar, Straus and Giroux. 10.1126/science.abm1652 34822293

[CR24] Haidt, J. (2012). *The righteous mind: Why good people are divided by politics and religion*. Penguin Books.

[CR25] Haidt, J., Koller, S. H., & Dias, M. (1993). Affect, culture, and morality, or is it wrong to eat your dog? *Journal of Personality and Social Psychology,**65*(4), 613–628. 10.1037/0022-3514.65.4.613 8229648 10.1037//0022-3514.65.4.613

[CR26] Hannon, E., & Lewens, T. (Eds.). (2018). *Why we disagree about human nature*. Oxford University Press.

[CR27] Hare, R. (1952). *The language of morals*. Clarendon Press; Oxford University Press.

[CR28] Joyce, R. (2006). *The evolution of morality. Life and mind*. MIT Press.

[CR29] Kirchin, S. (Ed.). (2013). *Thick concepts. First edition. Mind association occasional series*. Oxford University Press.

[CR30] Kitcher, P. (2011). *The ethical project*. Harvard University Press.

[CR31] Kronfeldner, M. (2018). *What’s left of human nature? A post-essentialist, pluralist, and interactive account of a contested concept. Life and Mind: Philosophical Issues in Biology and Psychology*. MIT Press.

[CR32] Kumar, V., & Campbell, R. (2022). *A better ape: The evolution of the moral mind and how it made us human*. Oxford University Press.

[CR33] Ludwig, D., & Ruphy, S. (2021). Scientific pluralism. In Zalta, E. (Ed), *The Stanford Encyclopedia of Philosophy*. https://plato.stanford.edu/cgi-bin/encyclopedia/archinfo.cgi?entry=scientific-pluralism

[CR34] Machery, E., & Mallon, R. (2010). Evolution of morality. In Doris, J. M. (Ed.), *The Moral Psychology Handbook* (pp. 3–46). Oxford University Press.

[CR35] Machery, E., & Stich, S. (2022). The moral/conventional distinction. In Zalta E. (Ed.), *Stanford Encyclopedia of Philosophy*. https://plato.stanford.edu/entries/moral-conventional/

[CR36] Müller, A. (2020). The social function of morality. In Hufendiek, R., James, D., & Van Riel, R. (Eds.), *Social Functions in Philosophy. Metaphysical, Normative, and Methodological Perspectives* (pp. 131–55). Routledge.

[CR37] Nichols, S. (2004). *Sentimental rules: On the natural foundations of moral judgment*. Oxford University Press.

[CR38] Nucci, L., & Turiel, E. (1978). Social interactions and the development of social concepts in preschool children. *Child Development,**49*(2), 400–407. 10.2307/1128704

[CR39] Nussbaum, M., & Sen, A. (1993). *The quality of life*. Oxford University Press.

[CR40] O’Neill, E., & Machery E. (2019). The normative sense: What Is Universal? What Varies? In Zimmerman, A., Jones, K., & Timmons M. (Eds.), *Routledge Handbook on Moral Epistemology* (pp. 38–57). Routledge.

[CR41] O’Neill, E. (2017). Kinds of norms. *Philosophy Compass,**12*(5), e12416.

[CR42] Plunkett, D. (2015). Which concepts should we use?: metalinguistic negotiations and the methodology of philosophy. *Inquiry,**58*(7), 828–874.

[CR43] Plunkett, D., & Sundell, T. (2013). Disagreement and the semantics of normative and evaluative terms. *Philosophers’ Imprint,**13*(23), 1–37.

[CR44] Prinz, J. (2007). *The emotional construction of morals*. Oxford University Press.

[CR45] Reichlin, M. (2024). The domain of morality. *Philosophia,**52*(3), 717–737. 10.1007/s11406-024-00750-4

[CR46] Roughley, N. (2018). From shared intentionality to moral obligation? some worries. *Philosophical Psychology,**31*(5), 736–754. 10.1080/09515089.2018.1486610

[CR47] Shweder, R., Much, N., Mahapatra, M., & Park, L. (1997). The ‘Big Three’ of morality (Autonomy, Community, Divinity) and the ‘Big Three’ explanations of suffering. In Brandt, A., & Rozin, P. (Eds.), *Morality and Health* (pp. 119–169). Routledge.

[CR48] Sinnott-Armstrong, W., & Miller, K. (Eds.). (2007). *Moral psychology: The evolution of morality: Adaptations and innateness*. The MIT Press. 10.7551/mitpress/7481.001.0001

[CR49] Sinnott-Armstrong, W., & Wheatley, T. (2014). Are moral judgments unified? *Philosophical Psychology,**27*(4), 451–474. 10.1080/09515089.2012.736075

[CR50] Smith, M. (1995). *The moral problem. Philosophical theory*. Blackwell.

[CR51] Smyth, N. (2017). The function of morality. *Philosophical Studies,**174*(5), 1127–1144. 10.1007/s11098-016-0746-8

[CR52] Southwood, N. (2011). The moral/conventional distinction. *Mind,**120*(479), 761–802. 10.1093/mind/fzr048

[CR53] Stich, S. (2019). The quest for the boundaries of morality. In Zimmerman, A., Jones, K., & Timmons M. (Eds.), *The Routledge Handbook of Moral Epistemology* (pp. 15–37). Routledge.

[CR54] Tomasello, M. (2016). *A natural history of human morality*. Harvard University Press.

[CR55] Turiel, E. (1983). *The development of social knowledge: Morality and convention. Cambridge studies in social and emotional development*. Cambridge University Press.

[CR56] Westra, E., & Andrews, K. (2022). A pluralistic framework for the psychology of norms. *Biology & Philosophy,**37*(5), 1–30. 10.1007/s10539-022-09871-0

[CR57] Williams, B. (1985). *Ethics and the limits of philosophy*. Oxford University Press.

[CR58] Young, L., & Dungan, J. (2012). Where in the brain is morality? everywhere and maybe nowhere. *Social Neuroscience,**7*(1), 1–10. 10.1080/17470919.2011.569146 21590587 10.1080/17470919.2011.569146

